# First study on molecular identification of *Anaplasma ovis* in sheep in southern Kazakhstan

**DOI:** 10.14202/vetworld.2025.67-75

**Published:** 2025-01-09

**Authors:** Alexandr Ostrovskii, Madina Kadyrova, Nurdina Yerzhanova, Dinara Kamalova, Amirkhan Kassen, Nailya Tursunbay, Alexandr Shevtsov, Christian Bauer, Kassym Mukanov

**Affiliations:** 1 National Center for Biotechnology, 01000, Astana, Kazakhstan; 2 Institute of Parasitology, Justus Liebig University Giessen, 35392, Giessen, Germany; 3 Department of Veterinary Medicine, S. Seifullin Kazakh Agro Technical Research University, 010011, Astana, Kazakhstan

**Keywords:** *Anaplasma ovis*, Kazakhstan, polymerase chain reaction, sequencing, sheep

## Abstract

**Background and Aim:**

Anaplasmosis in small ruminants is a tick-borne infection caused mainly by the obligate intraerythrocytic bacterium *Anaplasma ovis*. It is usually subclinical, with persistent infection in affected animals, but acute disease can occur, particularly in young animals. The pathogen is widespread in Central Asia and neighboring regions. In Kazakhstan, the infection was first detected in 1929. However, until now, diagnosis in the country has been based on traditional microscopic examination of blood smears. There were no reliable data on the prevalence and genetic diversity of *Anaplasma* spp. in sheep in Kazakhstan. This study aimed to determine the occurrence of *Anaplasma* spp. infection in sheep in southern Kazakhstan, a high-risk region for tick-borne diseases, using PCR and to identify the species by sequencing.

**Materials and Methods:**

A cross-sectional study was conducted on apparently healthy adult ewes from 77 settlements in 34 districts of Kyzylorda, Turkistan, Zhambyl, Almaty, and Jetisu, southern Kazakhstan. A total of 2553 whole blood samples collected in midsummer 2022 and 2023 were analyzed for *Anaplasma* spp. using polymerase chain reaction targeting the 404 bp *groEL* gene fragment. The amplification products from the 441 positive samples were sequenced using the Sanger sequencing method. Phylogenetic analysis of the obtained sequences was performed using the maximum likelihood model.

**Results:**

Overall, 1017/2553 (39.8%; 95% confidence interval: 37.9%-41.7%) ewes tested were positive for *Anaplasma* spp. Positive animals were found in 68/77 (88%) of the settlements from which samples were taken. The percentage of *Anaplasma* spp.-positive ewes varied significantly from 21.3% to 50.1% in the provinces. Altitude <500 m above sea level was identified as a risk factor for *Anaplasma* infection. All amplification products were identified as *A. ovis* through sequencing. Phylogenetic analysis of the *groEL* gene fragment sequences revealed the presence of two *A. ovis* genotypes; one was 100% identical to sequences from isolates from China and the other was >99.5% identical to isolates from Africa, Cyprus, and China.

**Conclusion:**

This first molecular study revealed a widespread of *A*. *ovis* infection in adult ewes in southern Kazakhstan. Altitude <500 m was identified as a risk factor. Therefore, clinical cases associated with *A. ovis* are expected in this region, especially in young animals. Future studies are needed to determine the clinical and economic impact of anaplasmosis on sheep production in the country, to investigate seasonal patterns of infection, and to identify tick species or other arthropods that act as local vectors. This information is useful for developing possible control measures and evaluating their effectiveness.

## INTRODUCTION

Ovine anaplasmosis is a tick-borne infection caused mainly by *Anaplasma ovis* Lestoquard, 1924 (Rickettsiales: Anaplasmataceae) [1]. In addition to *A. ovis*, infections with other *Anaplasma* species, such as *Anaplasma marginale*, *Anaplasma capra*, *Anaplasma bovis*, and *Anaplasma phagocytophilum* have been reported in sheep [2–7]. *A. ovis* is also commonly found in goats and occasionally in cattle, deer species, and reindeer [8–13]. Infections in small ruminants have been reported in Africa [1], the Americas [1], Asia [8, 14], and central and southern Europe [1, 11, 13, 14]. The primary vectors of *A. ovis* are thought to be hard ticks of the genus *Dermacentor* (especially *Dermacentor marginatus*), *Rhipicephalus* (e.g., *Rhipicephalus bursa* and *Rhipicephalus turanicus*), and others, although the vector competence of many species has not been proven [9, 15–18]. The pathogen is ingested by a suitable tick species during a blood meal. As described for the related species *A. marginale* [19], *A. ovis* probably replicates in ticks and invades its salivary glands. After the tick has molted to the next stage, the pathogen can be transmitted to a vertebrate host during a blood meal (transstadial transmission). Mechanical transmission by hematophagous insects, such as *Melophagus ovinus*, in which *A. ovis* DNA has been found [20, 21], appears to be possible but is thought to be of minor importance in spreading infection.

*A. ovis* is an obligate intraerythrocytic bacterium found in vertebrate hosts. The infection is usually subclinical in small ruminants [1]. However, acute disease may occur, particularly in lambs, in *A. ovis*-free animals introduced into an endemic area from non-endemic areas, or in chronically infected animals under stress. Clinical signs include fever, severe anemia, hemoglobinuria, weakness, weight loss, and reduced milk production [22–26]. Oxytetracycline or tetracycline hydrochloride can be used to treat acute clinical cases, but animals remain persistently infected after recovery [14]. They serve as reservoirs for pathogens and a source of further spread [22, 27]. The pathogen is widespread in Central Asia and neighboring regions. For example, the overall occurrence in sheep determined by molecular methods was 22% in Kyrgyzstan [4], 54% in north-eastern Iran [8], 56%–75% in Siberia [28, 29], 40%–64% in north-western China [24, 30], and 69% in Mongolia [31]. Ovine anaplasmosis was first diagnosed in 1929 in the Uralsk region of the former Soviet Union, located in the north-western part of present-day Kazakhstan [32]. Until now, the diagnosis of anaplasmosis in the country has been based on traditional microscopic examination of blood smears [33]. However, microscopy requires considerable experience, has low diagnostic sensitivity, especially in persistently infected animals, and cannot distinguish between intraerythrocytic *Anaplasma* spp. and their genotypes. Serological methods (e.g., enzyme-linked immunosorbent assay) for the detection of specific antibodies are unsuitable for genotype identification. Instead, molecular methods are more sensitive and reliable for genotyping [1, 8, 16, 26].

There were no reliable data on the prevalence and genetic diversity of *Anaplasma* spp. in small ruminants in Kazakhstan, a country with approximately 19,483,000 sheep and 2,300,000 goats [34]. This study aimed to determine the occurrence of *Anaplasma* spp. infection in sheep in southern Kazakhstan, a high-risk region for tick-borne diseases, using polymerase chain reaction (PCR) and to identify the species by sequencing.

## MATERIALS AND METHODS

### Ethical approval and Informed consent

The study was approved by the Ethics Committee of the National Center for Biotechnology LLP, Kazakhstan (Protocol No. 2, dated April 4, 2022). Sheep owners provided oral consent for blood sampling.

### Study period and location

**Figure 1:**
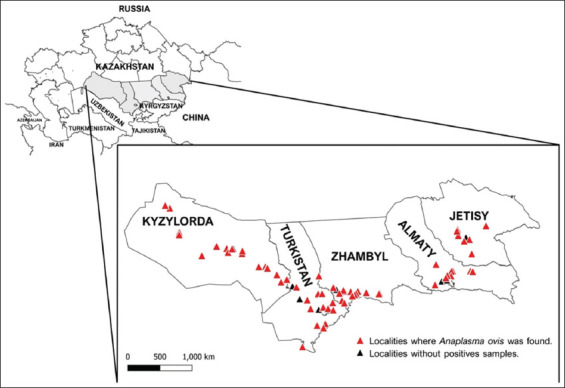
Map of Kazakhstan showing sampling locations in the five southern provinces and *Anaplasma* spp. Polymerase chain reaction (PCR)-positive and PCR-negative locations [Source: The map was generated using QGIS version 3.34 (https://www.qgis.org)].

The study was conducted from July 2022 to August 2023. The cross-sectional study was conducted on sheep from 77 settlements in 34 districts of the five southern provinces of Kyzylorda, Turkistan, Zhambyl, Almaty, and Jetisu, Kazakhstan (Figure 1). According to the official veterinary census, the number of sheep kept in these provinces at the end of 2023 was approximately 12,120,000, or about 62% of the country’s total sheep population. The majority (about 91%–98%) of sheep in these provinces are kept in private households and on small farms for slaughter [34].

The sampled settlements varied in altitude from approximately 50–1,250 m above sea level (masl) (Supplementary data). Depending on their location, the sampling sites were characterized by a cold semi-arid climate (“Bsk” climate zone according to the Köppen-Geiger classification [35]: Kyzylorda, parts of Turkistan and Zhambyl), a temperate climate with hot dry summers (“Csa”: Parts of Turkistan), a continental climate with warm to hot dry summers (“Dsa” or “Dsb”: Parts of Zhambyl), or a humid continental climate with long winters and warm to hot summers but no dry season (“Dfa” or “Dfb”: Almaty, Jetisu).

### Sampling

Flocks with ewes at least 3 years old were selected for the study. On the day of sampling, all sheep at a specific location were brought together, and a sample was taken from the first approximately 10% of ewes reached. The ewes sampled were of a local breed. They were not specifically examined for ticks, but they appeared clinically healthy. Whole blood samples were collected in vacuum tubes containing ethylenediaminetetraacetic acid (EDTA). Samples were transported to the laboratory within 48 h at 4°C–8°C and immediately processed for DNA isolation. As some blood samples were unusable, 2553 samples were available for laboratory analysis (mostly samples 20–30/location; Supplementary data).

### DNA extraction

Before DNA extraction, blood samples were mixed with an erythrocyte lysis solution containing 1.5 M NH_4_Cl, 100 mM NaHCO_3_, 10 mM EDTA. Lysis was performed for 5 min at 20°C–22°C (room temperature)., followed by centrifugation at 13,000× *g* to remove the supernatant. DNA was isolated from the precipitates using a DNA/RNA-S-FACTOR kit (Vet Factor, Troitsk, Russia), according to the manufacturer’s instructions.

### PCR

All samples were tested for *Anaplasma* spp. by PCR targeting a 404-bp fragment of the heat shock protein *groEL* gene fragment using the primer pairs anapl_F-1393 (5’-aaggatggatayaaggtmatgaa-3’, forward) and anapl_R1852 (5’-cgcggwcaaactgcatac-3’, reverse) [36]. The PCR mix contained 300 nM of both forward and reverse primers, 0.2 mM of each dNTP, 2.5 mM MgCl_2_, 10 mM Tris-HCl (pH 9.0), 50 mM KCl, 0.1% Triton X-100, 2 U Taq DNA polymerase (Biolabmix, Novosibirsk, Russia), and 40 - 100 ng of DNA. The PCR reaction was performed using a Mastercycler ProS (Eppendorf, Hamburg, Germany) with the following protocol: Pre-denaturation for 5 min at 95°C, followed by 35 cycles of 30 s at 95°C, 40 s at 60°C, and 50 s at 72°C, with a final extension at 72°C for 5 min.

### Validation and preparation for sequencing

PCR products were detected by electrophoretic separation of fragments in a 1.5% agarose gel with ethidium bromide, followed by visualization using the GelDoc XR+ system (Bio-Rad, Hercules, USA). The amplicons were purified from the remaining components of the reaction mixture by binding to magnetic particles. The magnetic particles were washed 3 times with 3% HCl and then suspended at 1:49 in a binding buffer (20% polyethylene glycol, 2.5 M NaCl). The PCR products were mixed with 0.8× magnetic particle solution and vortexed at 1,800 rpm for 2 min. This was followed by 10 min of incubation at room temperature. The plate was then placed on a magnetic rack, the supernatant was removed, and the particles were washed twice with 70% ethanol. Elution was performed in 1× TE (AppliChem, Barcelona, Spain) at 60°C for 10 min.

### Sequencing and phylogenetic analyses

The amplification products from approximately 40% of the PCR-positive samples (Supplementary data) were sequenced to identify the *Anaplasma* spp. involved. Sanger sequencing of the 404 bp *groEL* gene fragment was performed using the BigDye Terminator v3.1 Cycle Sequencing Kit (Thermo Fisher Scientific, Vilnius, Lithuania), according to the manufacturer’s instructions. Separation and detection of the sequenced samples were performed using the 3730 series Genetic Analyzers (Applied Biosystems, Carlsbad, USA). The resulting sequences were assembled using SeqMan software (Lasergene, DNASTAR, Madison, USA) [37]. The ClustalW tool of the MEGA X software (https://www.megasoftware.net/) was used for multiple alignments. Phylogenetic analysis was performed by maximum likelihood using the Kimura 2-parameter model with discrete gamma distribution and invariant sites; five rate categories were used. Bootstrap support was calculated based on 100 replicates. MEGA X software (https://www.megasoftware.net/) was used to visualize and construct the phylogenetic tree [31].

### Statistical analysis

Exploratory data analysis was performed using the BIAS statistical software (version 9.05; Epsilon, Hochheim, Germany) [38]. The prevalence [39] of *Anaplasma* infection and its 95% confidence interval (CI) were calculated. Univariate analysis was performed using Chi-square test to compare occurrence at different settlement altitudes (grouped as < 200 m, 200–500 m, and > 500 m) for significance. For the latter, odds ratios (ORs) were calculated. Differences with p < 0.05 were considered statistically significant.

## RESULTS

**Figure 2:**
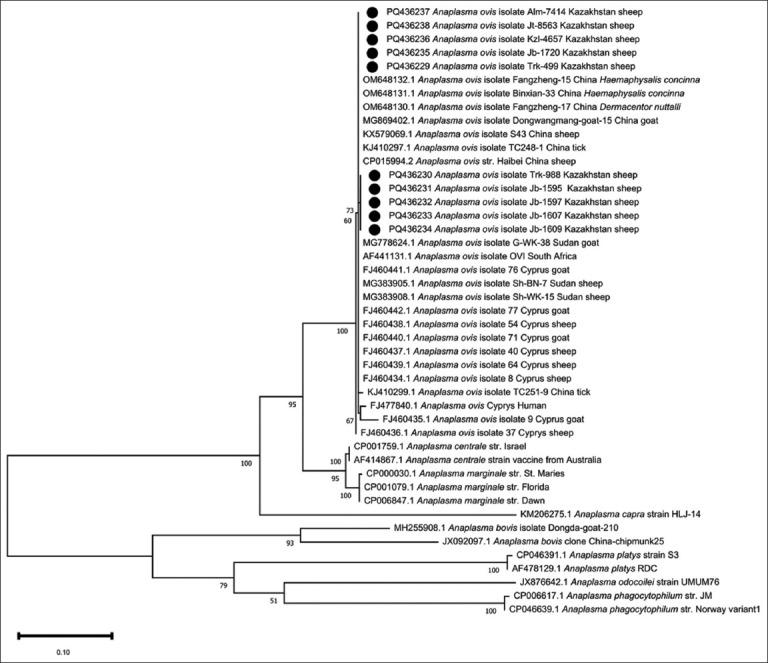
Phylogenetic tree of *Anaplasma ovis* and other *Anaplasma* spp. based on *groEL* gene fragment sequences (MEGA X software; https://www.megasoftware.net). Nodal support is indicated by bootstrap values in percent; the scale bar indicates the estimated numbers of nucleotide substitutions per position; species name, followed by country and host, if any. The solid circles indicate the sequences obtained in this study (for clarity, only one sequence of each genotype from each province sampled is included).

*Anaplasma* spp. infections were detected by *groEL* PCR in adult sheep in 68/77 (88%) 77 settlements from which samples were taken and in all five provinces (Figure 1). There was at least one negative settlement in each province. In total, 1017 (39.8%) of the 2553 ewes were positive. The percentage of positive ewes in the provinces varied from 21.3% to 50.1%, with highly significant differences (p < 0.0001) (Table 1). The percentage within positive settlements ranged from 3% to 100% (median: 50%; Supplementary data). The percentage of *Anaplasma* spp. DNA-positive ewes was associated with settlement altitude: It was significantly lower in settlements >500 masl (34.7%) than in those at lower altitudes (45.6%–45.7%). The chance of detecting positive ewes was 1.6 times higher in settlements <500 masl (OR: 1.6) than in those at higher altitudes (Table 2). Amplification products from 441 positive *Anaplasma* spp. samples were sequenced for specification, and all were identified as *A. ovis*. For the multiple alignments of amplification products performed with the ClustalW tool, reference *Anaplasma* spp. (*A. marginale*, *A. ovis*, *A. centrale*, *A. platys*, *A. bovis*, *A. capra*, *A. phagocytophilum*, and *A. odocoilei*) and all available *A. ovis* genotypes from GenBank as of September 2024 were used. Phylogenetic analysis revealed the presence of two genotypes (Figure 2): One genotype (GenBank accession numbers: PQ436229, PQ436235–PQ436238) was found in all five provinces. It was 100% identical to sequences from *A. ovis* isolates from China. The other genotype (GenBank accession numbers: PQ436230–PQ436234) was detected in only five sequenced samples from Turkistan and Zhambyl. It differed from the first genotype by one nucleotide and formed a separate subclade, sharing 99.75%, 99.5%, and 99.75% identity with *A. ovis* isolates from Africa, Cyprus, and China, respectively.

**Table 1:** *Anaplasma ovis* infection detected by polymerase chain reaction in adult ewes from settlements in southern Kazakhstan.

Sampling locations	No. of settlements	No. of settelements (%) with positive ewes	Ewes		% of positive ewes (95% CI)	p-value

			No. of tested	No. of positive		
Kyzylorda	20	19 (95)	623	312	50.1 (46.1–54.1)	<0.0001^1^
Turkistan	18	15 (83)	580	244	42.1 (38.0–46.2)	
Zhambyl	18	16 (89)	570	240	42.1 (38.0–46.3)	
Almaty	13	11 (85)	480	102	21.3 (17.7–25.2)	
Jetisu	8	7 (88)	300	119	39.7 (34.1–45.4)	
Total	77	68 (88)	2553	1017	39.8 (37.9–41.7)	

CI=Confidence interval.

1 Significance level of difference between provinces determined using the Chi-square test

**Table 2:** Association between settlement altitude and *Anaplasma ovis* infection in adult ewes from settlements in southern Kazakhstan.

Altitude (masl)	No. of settlements	No. of settlements (%) with positive ewes	Ewes		% of positive ewes (95% CI)	Odds ratio (95% CI)	p-value

			No. of tested	No. of positive			
<200	20	18 (90)	623	284	45.6 (41.6–49.6)^a^	1.6 (1.3–1.9)	<0.0001
200–500	20	18 (90)	580	265	45.7 (41.6–49.8)^a^	1.6 (1.3–1.9)	<0.0001
>500	37	32 (86)	1350	468	34.7 (32.1–37.3)^b^	Reference	

CI=Confidence interval. ^a,b^Values with different superscripts are significantly different (p < 0.0001)

## DISCUSSION

This is the first molecular study of ovine anaplasmosis in Kazakhstan. The percentage of ewes positive for *Anaplasma* spp. by PCR was 39.8%, and subsequent *groEL* gene fragment sequencing identified only *A. ovis*, confirming previous data on its presence in microscopically examined sheep from southern Kazakhstan [33]. These data should be interpreted in the context that the blood samples were collected from apparently healthy adult sheep in midsummer. During this sampling period, tick species present in southern Kazakhstan [30, 40–44], which are potential vectors of *A. ovis*, such as *D. marginatus* and *Rhipicephalus turanicus* [15–18], are still in the period of activity [15, 45]. Subclinical infections in *A. ovis-*positive sheep, particularly older animals, have been reported in many studies [2–4, 10, 46]. This indicates endemic stability in the region due to the development of immunity to the pathogen in older animals. Endemic stability is defined as a situation in which the relationship between the vertebrate host, pathogen, vector, and environment is that clinical disease is rare despite the presence of a large number of subclinically infected animals in the population [47]. In such epidemiological situations, clinical disease may occur, for example, in young animals that have not yet developed immunity, in pathogen-naive animals introduced into the region, or in chronically infected animals under stress [23, 24]. This suggests that, at least under certain conditions, *A. ovis* may cause clinical signs and economic losses in Kazakhstan. Therefore, it would be advisable to further investigate ovine anaplasmosis and its clinical and economic impact on Kazakhstan.

The provincial occurrence of *A. ovis* infection was very variable in the present study: In the Almaty region, which has a humid continental climate with long winters and warm/hot summers but no dry season, the proportion of positive animals was less than half that in the Kyzylorda region, which has a cold, semi-arid steppe climate (Table 1). Altitude <500 masl was identified as a risk factor for *Anaplasma* infection (Table 2). In Kazakhstan, the overall percentage of *Anaplasma* spp.-positive ewes was much higher (39.8%) than in neighboring Kyrgyzstan (22% [4]) but lower than in Türkiye (67% [3]) using *groEL* PCR. Studies using PCR for the *Anaplasma* major surface protein 4 (*msp4*) gene or the *16S ribosomal RNA* (*16S rRNA*) gene have also shown large variations in prevalence within and between Asian countries, such as 0%–94% in neighboring north-western China [2, 5], 7%–93% in Mongolia [10], 5%–100% in Siberia [29, 29], 1%–70% in Iran [48], 59%–67% in Iraq [1, 49], and 10%–88% in Türkiye [1, 46]. In addition to *A. ovis*, other *Anaplasma* spp., such as *A. marginale*, *A. capra*, *A. bovis*, and *A. phagocytophilum*, have been detected in sheep in some regions [2–5, 7]. All these regional differences in this and other studies, as well as differences between countries, can be explained by differences in topography, vegetation, and local microclimate in the areas studied. These factors strongly affect the local species composition of tick populations and the availability of suitable tick species as vectors and, of course, the resulting infection with *Anaplasma* spp. and other tick-borne pathogens [50]. In addition, the sheep tested (breed, age, and health status), parasite control, type of husbandry, and sampling time may have varied between studies and, thus, could have influenced the results. For example, in an Iranian study by Noaman and Sazmand [48], a short distance between neighboring farms was a risk factor for *A. ovis* infection, and older sheep and foreign breeds were significantly more likely to be *A. ovis*-positive than younger animals and native breeds. Therefore, it seems to make little sense to directly compare the results of different studies without considering these factors.

In our study, a fragment of the *groEL* gene isolated from *Anaplasma* spp. was used as the PCR target. The *msp4* and *16S rRNA* genes [5, 51] are often used as targets to analyze genetic diversity in *A. ovis*, but the gene best suited for intraspecific differentiation of isolates is the major surface protein 1a (*Msp1a*) gene, which is under strong immune selection pressure [52, 53]. The *groEL* gene may be more informative than the *msp4* and *16S rRNA* genes for studying genetic diversity across hosts and geographical regions [54, 55]. For example, in a phylogenetic analysis of *A. ovis* from Tunisia, *groEL* fragment sequences from small rodents and infesting ticks clustered separately from those detected in camels, and several new variants characteristic of the region were found [55].

Phylogenetic analysis of a 900 bp *groEL* gene fragment from animals in Cyprus allowed differentiation of 10 genotypes [56], whereas in the present study, analysis of a 404 bp *groEL* gene fragment identified only two of the Cypriot genotypes (Figure 2). This suggests that the 404 bp region was not sufficiently informative for the identification of *A. ovis* isolates, probably due to its high conservatism and the insufficient geographical distance of the isolates. Nevertheless, we were able to identify two genotypes, one of which was completely identical and the other more than 99.5% identical to GenBank reference sequences from other countries. Understanding the phylogenetic relationships between *A. ovis* isolates is key to analyzing intraspecific diversity in different regions, which may improve the prevention and control of anaplasmosis, for example, by reducing the immigration and spread of this pathogen.

## CONCLUSION

This study represents the first molecular investigation into *A. ovis* infections in sheep in southern Kazakhstan, revealing a high percentage of *Anaplasma* spp.-positive adult ewes at 39.8% and identifying altitude as a significant risk factor. The presence of two *A. ovis* genotypes, with similarities to isolates from China, Africa, and Cyprus, underscores the interconnected nature of infectious diseases and their global epidemiological patterns. These findings highlight the endemic stability in the region and the potential for economic and clinical impacts, especially in young or immunologically naive sheep populations.

Future research should focus on assessing the clinical and economic burdens of anaplasmosis on Kazakhstan’s livestock industry, particularly on sheep productivity and welfare. In addition, investigations into the seasonal dynamics of infection and the identification of specific vector species are crucial for understanding transmission ecology. Expanding phylogenetic analyses to include more diverse gene regions could provide deeper insights into genetic diversity and adaptation of *A. ovis* in different ecological niches. Developing region-specific tick and vector management strategies, alongside molecular diagnostics and vaccination programs, will be critical to mitigate the impact of anaplasmosis in southern Kazakhstan and beyond.

## DATA AVAILABILITY

The supplementary data can be available from the corresponding author upon a request.

## AUTHORS’ CONTRIBUTIONS

AO: Sequencing and drafted the manuscript. MK, NY, and DK: PCR. AK and NT: DNA extraction. AS: Data analysis and curation. CB: Data interpretation, statistical analysis, and critical review and revised the manuscript. KM: Project design, supervision, and administration. All authors have read and approved the final manuscript.
